# Nomogram-based risk prediction of macrosomia: a case-control study

**DOI:** 10.1186/s12884-022-04706-y

**Published:** 2022-05-05

**Authors:** Jing Du, Xiaomei Zhang, Sanbao Chai, Xin Zhao, Jianbin Sun, Ning Yuan, Xiaofeng Yu, Qiaoling Zhang

**Affiliations:** Department of Endocrinology and metabolism, Peking University International Hospital, No. 1 Life Garden Road Zhongguancun Life Science Garden Changping District, Beijing, 102206 China

**Keywords:** Macrosomia, Nomogram, Screening, Risk factor

## Abstract

**Background:**

Macrosomia is closely associated with poor maternal and fetal outcome. But there is short of studies on the risk of macrosomia in early pregnancy. The purpose of this study is to establish a nomogram for predicting macrosomia in the first trimester.

**Methods:**

A case-control study involving 1549 pregnant women was performed. According to the birth weight of newborn, the subjects were divided into macrosomia group and non-macrosomia group. The risk factors for macrosomia in early pregnancy were analyzed by multivariate logistic regression. A nomogram was used to predict the risk of macrosomia.

**Results:**

The prevalence of macrosomia was 6.13% (95/1549) in our hospital. Multivariate logistic regression analysis showed that prepregnancy overweight (OR: 2.13 95% CI: 1.18–3.83)/obesity (OR: 3.54, 95% CI: 1.56–8.04), multiparity (OR:1.88, 95% CI: 1.16–3.04), the history of macrosomia (OR: 36.97, 95% CI: 19.90–68.67), the history of GDM/DM (OR: 2.29, 95% CI: 1.31–3.98), the high levels of HbA1c (OR: 1.76, 95% CI: 1.00–3.10) and TC (OR: 1.36, 95% CI: 1.00–1.84) in the first trimester were the risk factors of macrosomia. The area under ROC (the receiver operating characteristic) curve of the nomogram model was 0.807 (95% CI: 0.755–0.859). The sensitivity and specificity of the model were 0.716 and 0.777, respectively.

**Conclusion:**

The nomogram model provides an effective mothed for clinicians to predict macrosomia in the first trimester.

## Background

Macrosomia is one of the most common neonatal adverse outcomes, which is defined as the absolute birth weight of newborn > 4000 g or 4500 g, regardless of the gestational age [1]. Recent studies have showen that macrosomia increases adverse maternal and fetal outcomes. For pregnant women, delivery of macrosomia is associated with significantly elevated risks of cesarean section, prolonged labor, postpartum hemorrhage, chorioamnionitis, soft birth canal injury, even uterus and bladder rupture [2–6]. For the newborns, macrosomia increases the risks of shoulder dystocia, clavicle fractures, brachial plexus injury, respiratory distress, meconium aspiration, perinatal infection, hypoglycemia, polycythemia, hypoxic-ischemic encephalopathy and increases the need for admission to neonatal intensive care unit [3–5, 7, 8]. In addition, macrosomia also causes more health problems such as diabetes mellitus, overweight/obesity, high blood pressure, cardiovascular disease and other chronic diseases later in life [9–11]. Predicting macrosomia and taking proper interventions during early stage of pregnancy can avoid or decrease adverse complications. Macrosomia can only be diagnosed accurately by weighing the newborn after birth, but the opportunity of early intervention is lost. Currently, the prenatal estimation of fetal weight is carried out by maternal physical examination or ultrasonographic measurement. However, these methods are based on maternal and fetal data during the late stage of pregnancy, and the accuracies are not very high [12, 13]. Hence, establishing a simple, noninvasive, practical, accurate model for predicting the risk of macrosomia in the early stage of pregnancy is of great importance. In this study, we attempted to set up a predictive model of macrosomia based on clinical indicators in first trimester, which enables early identification and preventation of macrosomia.

## Materials and methods

### Subjects

**Fig. 1:**
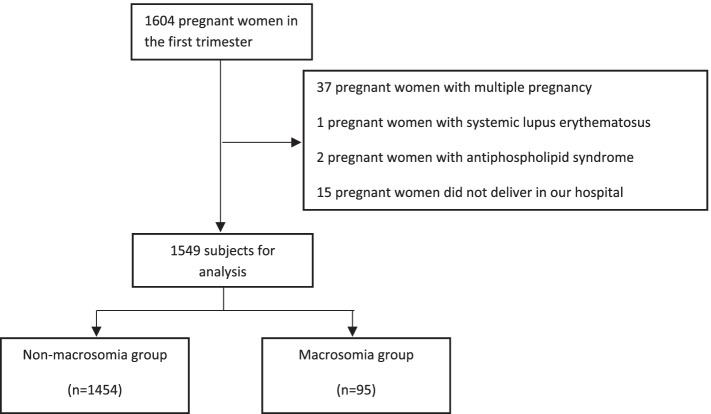
Flow chart of subject selection

This was a case-control study. Pregnant women admitted to the Department of Gynaecology and Obstetrics of Peking University International Hospital from December 2017 to June 2019 were enrolled to this study. Inclusion criterias: (1) subjects’ age ≥ 18 years old; (2) the gestational weeks < 12 weeks diagnosed by LMP (last menstrual period) and HCG (human chorionic gonadotropin); (3) single pregnancy; (4) planning to undergo examine and give birth at our hospital. Exclusion criterias: (1) multiple pregnancy; (2) subjects with cardiovascular and cerebrovascular diseases, respiratory diseases, liver and kidney diseases, hematological diseases, autoimmune diseases (e.g., systemic lupus erythematosus, antiphospholipid syndrome), and tumors; (3) subjects who did not deliver in our hospital. Finally, a total of 1549 subjects with complete data were recruited in this study (Fig. 1).

## Methods

Demographic and medical data were recorded, including age, parity, gestational week, prepregnancy weight, past medical history [history of macrosomia, GDM (gestational diabetes mellitus) or DM (diabetes mellitus), HDP (hypertensive disorders of pregnancy), etc.]. All patients underwent anthropometric assessment, including height and blood pressure. BMI (Body mass index) was calculated: BMI = weight (kg)/square of height (m^2^).

Venous blood samples were drawn from pregnant women (gestational 5–12 weeks) after fasting for 8 hours to evaluate ALT (alanine aminotransferase), AST (aspartate aminotransferase), ALB (albumin), TC (total cholesterol), TG (triglyceride), LDL-C (low-density lipoprotein cholesterol), HDL-C (high-density lipoprotein cholesterol), Scr (serum creatinine), uric acid (UA), HbA1c (glycosylated hemoglobin), fasting plasma glucose (FPG), CRP (C-reactive protein), FT3 (free triiodothyronine), TSH (thyroid stimulating hormone), and FT4 (free thyroxine).

All subjects were followed up regularly during pregnancy until delivery. Gestational weight gain of pregnant women and birth weight of newborns were measured and recorded by well-trained nurses.

### Definitions

Macrosomia was defined as an absolute birth weight over 4000 g [14]. Overweight was defined as BMI 24-28 kg/m^2^, obesity was defined as BMI ≥ 28 kg/m^2^ [15]. GDM was diagnosed based on the guidelines of the International Association of Diabetes and Pregnancy Study Groups as fasting plasma glucose (FPG) ≥ 5.1 mmol/L, and/or 1-hour plasma glucose (1hPG) ≥ 10.0 mmol/L, and/or 2-hour plasma glucose (2hPG) ≥ 8.5 mmol/L in a 75 g oral glucose tolerance test (OGTT) [16]. DM was defined based on the criteria presented by the World Health Organization (WHO) in 1999 as FPG ≥ 7.0 mmol/L and/or 2hPG ≥ 11.1 mmol/L in OGTT. HDP included gestational hypertension, preeclampsia-eclampsia, chronic hypertension (of any cause diagnosed before 20 weeks of gestation), and chronic hypertension with preeclampsia superimposed [17].

### Statistical analysis

Statistical analyses were performed with SPSS 20.0 software (provided by IBM, Armonk, NY, USA). Normality was assessed with the Shapiro-Wilk test. Continuous variables were expressed as mean ± standard deviation or median (Q1, Q3). Categorical variables were expressed as absolute numbers and percentages. To compare the differences between the two groups, Chi-square test, Mann–Whitney U test or independent t-test was employed as appropriate. Logistic regression with a forward stepwise process was used to analyze the risk factors of macrosomia. *P* <  0.05 indicated significant differences.

Statistical packages R (http://www.R-project.org) and EmpowerStats (www.empowerstats.com, X&Y Solutions, Inc., Boston, MA) were used to establish and validate the nomogram. In this study, the nomogram was internally validated by using 500 bootstrap resamplings. We created a receiver operating characteristic (ROC) curve and calculated the area under the ROC curve (AUC) to assess the discriminatory ability of the nomogram. The value of AUC closer to 1 implied a better predictive accuracy.

## Results

### Subjects’ characteristics of the case control study

Among the 1549 pregnant women, 95 mothers gave birth to the newborn weighed more than 4000 g. The rate of macrosomia was 6.13%. There was no significant difference in the mean age and the proportion of pregnant women older than 35-year-old between the macrosomia group and the non- macrosomia group (*P* > 0.05). The prepregnancy BMI and weight gain during pregnancy in the macrosomia group were higher than that in the non-macrosomia group, and the proportions of overweight and obese participants before pregnancy were higher in the macrosomia group compared with the non-macrosomia group (*P* <  0.05). Compared to the non-macrosomia group, more participants were multiparas and with the history of macrosomia and GDM/DM in the macrosomia group (*P* <  0.05). In the first trimester, the levels of TC, TG, HbA1c and CRP of pregnant women in the macrosomia group were higher than those in the non-macrosomia group (*P* <  0.05), whereas, there was no significant difference on the levels of SBP, DBP, ALT, AST, ALB, HDL, LDL, FPG, SCr, UA, FT4, FT3 and TSH between the two groups (*P* > 0.05) (Table 1).

**Table 1:** Comparison of maternal characteristics between the non-macrosomia group and the macrosomia group

Index	Non-macrosomia group (*n* = 1454)	Macrosomia group (*n* = 95)	*P* value
Age (years)	30.91 ± 3.64	31.46 ± 3.65	0.148
< 25	30 (2.1%)	1 (1.1%)	
25–34	1175 (80.8%)	73 (76.8%)	
≥ 35	249 (17.1%)	21 (22.1%)	0.387
Prepregnancy BMI (kg/m^2^)	21.84 ± 2.97	23.13 ± 3.12	< 0.001
< 24	1169 (80.4%)	60 (63.2%)	
24–28	225 (15.5%)	22 (23.2%)	
≥ 28	60 (4.1%)	13 (13.7%)	< 0.001
Gestational weight gain (kg)	12.53 ± 5.62	14.97 ± 5.09	< 0.001
Parity			
0	906 (62.3%)	46 (48.4%)	
≥ 1	548 (37.7%)	49 (51.6%)	0.007
History of macrosomia			
Yes	30 (2.1%)	34 (35.8%)	
No	1424 (97.9%)	61 (64.2%)	< 0.001
History of GDM/DM			
Yes	278 (19.1%)	29 (30.5%)	
No	1176 (80.9%)	66 (69.5%)	0.007
History of HDP			
Yes	18 (1.2%)	3 (3.2%)	
No	1436 (98.8%)	92 (96.8%)	0.267
SBP (mmHg)	109.73 ± 9.97	111.64 ± 9.43	0.071
DBP (mmHg)	65.92 ± 9.15	67.53 ± 9.85	0.100
ALT (U/L)	17.01 ± 3.59	17.92 ± 8.51	0.303
AST (U/L)	18.27 ± 8.96	19.08 ± 15.27	0.416
ALB (g/L)	44.18 ± 2.50	43.72 ± 2.28	0.085
TC (mmol/L)	3.93 ± 0.70	4.13 ± 0.91	0.046
TG (mmol/L)	0.99 ± 0.60	1.18 ± 0.69	0.010
HDL-C (mmol/L)	1.42 ± 0.36	1.41 ± 0.26	0.805
LDL-C (mmol/L)	2.55 ± 1.07	2.23 ± 0.62	0.768
SCr (umol/L)	49.97 ± 12.41	49.72 ± 6.41	0.845
UA (umol/L)	213.58 ± 50.81	222.13 ± 54.16	0.114
HbA1c (%)	5.12 ± 0.27	5.27 ± 0.71	0.047
FPG (mmol/L)	4.91 ± 0.27	5.00 ± 0.59	0.151
CRP (mg/L)	2.00 ± 2.30	3.05 ± 3.56	0.005
FT4 (pmol/L)	17.45 ± 7.65	16.51 ± 2.51	0.232
FT3 (pmol/L)	4.79 ± 2.29	4.65 ± 0.57	0.545
TSH (mU/L)	1.89 ± 1.72	1.92 ± 1.33	0.852

*BMI* Body mass index, *GDM* Gestational diabetes mellitus, *DM* Diabetes mellitus, *HDP* Hypertensive disorders of pregnancy, *SBP* Systolic blood pressure, *DBP* Diastolic blood pressure, *ALT* Alanine aminotransferase, *AST* aspartate aminotransferase, *ALB* Albumin, *TC* Total cholesterol, *TG* Triglycerides, *HDL-C* High-density lipoprotein cholesterol, *LDL-C* Low-density lipoprotein cholesterol, *SCr* Serum creatinine, *UA* Uric acid, *HbA1c* Glycosylated hemoglobin, *FPG* Fasting plasma glucose, *CRP* C-reactive protein, *FT4* Free thyroxine, *FT3* Free triiodothyronine, *TSH* Thyroid stimulating hormone

### Logistic regression analysis of the risk factors of macrosomia

Multiple logistic regression analysis was performed with delivery of macrosomia as the dependent variable, and with statistically significant variables from univariate regression analysis included gestational weight gain, prepregnancy BMI, history of GDM/DM, parity, history of macrosomia, HbA1c, TC, TG and CRP as independent variables. After adjusting for the weight gain during pregnancy, macrosomia was significantly associated with prepregnancy overweight (OR: 2.13, 95% CI: 1.18–3.83)/obesity (OR: 3.54, 95% CI: 1.56–8.04), multiparity (OR:1.88, 95% CI: 1.16–3.04), the history of macrosomia (OR: 36.97, 95% CI: 19.90–68.67), the history of GDM/DM (OR: 2.29, 95% CI: 1.31–3.98), the levels of HbA1c (OR: 1.76, 95% CI: 1.00–3.10) and TC (OR: 1.36, 95% CI: 1.00–1.84) (Table 2).

**Table 2:** Multivariate logistic regression analysis of risk factors of macrosomia

	β	SE.	Wald χ2	OR (95%CI)	*P* value
**Prepregnancy BMI (kg/m2)**					
** < 24**	Ref.				
** 24–28**	0.75	0.30	6.33	2.13 (1.18, 3.83)	0.012
** ≥ 28**	1.26	0.42	9.09	3.54 (1.56, 8.04)	0.003
**Parity**					
** 0**	Ref.				
** ≥ 1**	0.63	0.25	6.56	1.88 (1.16, 3.04)	0.010
**History of macrosomia**					
** No**	**Ref.**				
** Yes**	3.61	0.32	130.56	36.97 (19.90, 68.67)	< 0.001
**History of GDM/DM**					
** No**	Ref.				
** Yes**	0.83	0.28	8.56	2.29 (1.31, 3.98)	0.003
**HbA1c (%)**	0.57	0.29	3.90	1.76 (1.00, 3.10)	0.048
**TC (mmol/L)**	0.31	0.16	3.94	1.36 (1.00, 1.84)	0.047

*BMI* Body mass index, *GDM* Gestational diabetes mellitus, *DM* Diabetes mellitus, *HbA1c* Glycosylated hemoglobin, *TC *Total cholesterol

### Establishment and validation of the nomogram for macrosomia

**Fig. 2:**
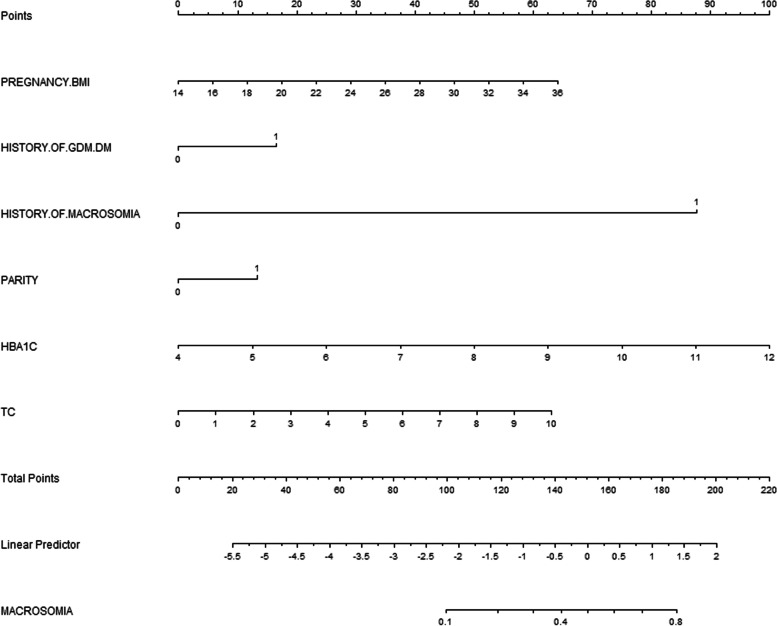
Nomogram for predicting macrosomia in first trimester. Instructions: The point score of each risk factor can be calculated separately by reading the score above the factor vertically. Then, the points from each variable value were summed. The sum on the total points scale was located and vertically projected onto the bottom axis, and then a personalized macrosomia risk was obtained

**Fig. 3:**
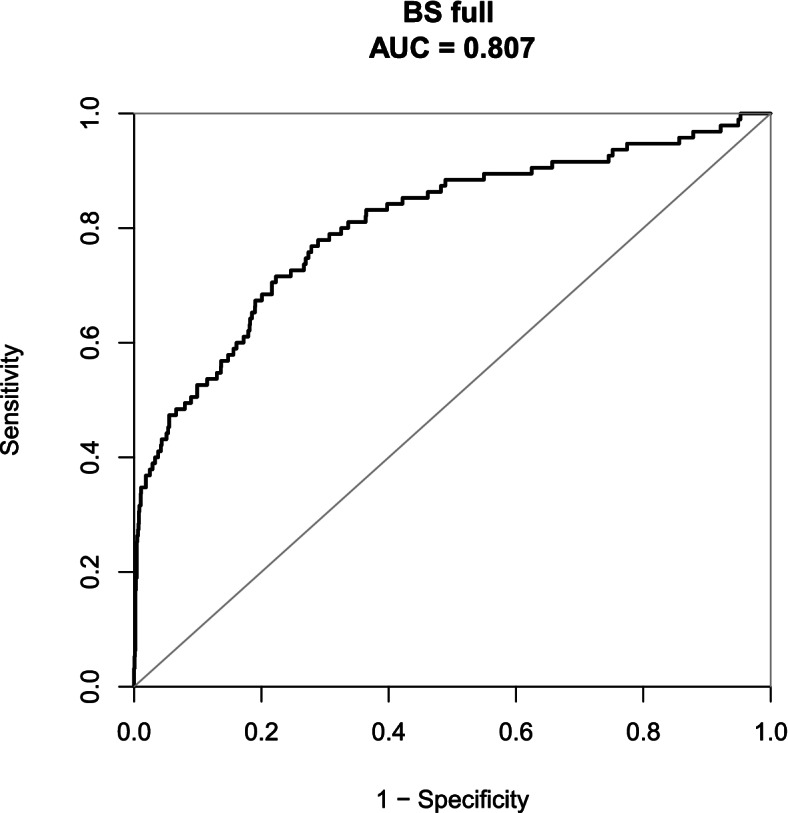
The ROC curve of the nomogram model for macrosomia

In the study, the nomogram for predicting the risk of macrosomia was established based on the risk factors identified by the multivariate logistic regression analysis including prepregnancy BMI, parity, history of macrosomia, history of GDM/DM, first-trimester HbA1 and TC levels (Fig. 2). The AUC of the nomogram model for macrosomia was 0.807 (95% CI: 0.755–0.859). The sensitivity and specificity were 0.716 and 0.777, respectively (Fig. 3).

## Discussion

The birth weight of newborn and the incidence of macrosomia are both increasing along with economic improvement recently. In this study, prevalence of macrosomia was 6.13%, which was close to 5.96% reported in the tertiary hospitals and lower than that in the total prevalence (7.069%) in beijng [18]. This is comparable to the United states (7.8%) and requires intervention [19].

Macrosomia is closely related to a variety of maternal factors, such as genetic factors, environment, and healthy condition. Previous studies have shown that prepregnancy BMI, excessive weight gain during pregnancy, and preexisting GDM/DM are independent risk factors for macrosomia. GDM/DM, overweight/obesity and excess gestational weight gain have more metabolic characteristics such as increased insulin resistance, hyperglycemia, and hyperinsulinemia, which play important roles in macrosomia [20–23] This study found that prepregnancy BMI, weight gain during pregnancy, the proportion of preexisting GDM/DM in the macrosomia group were significantly higher than those in the non-macrosomia group (*P* < 0.05). The result of multivariate logistic regression showed the risk of macrosomia were significantly and independently associated with prepregnancy overweight /obesity, the history of GDM/DM and weight gain during pregnancy, which was consistent with the above findings.

A multi-center, cross-sectional survey involving 101,723 singleton term infants showed that risk of macrosomia was positively associated with maternal age, parity [24]. In this study, the mean age and the frequency of pregnant women ≥35-year-old in the macrosomia group were higher than those in the non- macrosomia group, but the difference was not statistically significant. And there was no association between age and macrosomia by univariate logistic regression analysis (*P* > 0.05). Multiparas in the macrosomia group was observed more than that in the non- macrosomia group, and the risk of macrosomia in multiparas was 1.8 times higher than that in primiparas(*P* < 0.05). These results were not completely consistent with the previous studies.

Data from a prospective cohort study including 54,371 singleton pregnancies at 12 centers in the US showed that women who had delivered a macrosomic newborn in the past had a higher risk to have another macrosomia in the subsequent pregnancy with the recurrence rate of 23.2% (95% CI: 21.2–25.2%), and the number of prior macrosomic infants was positively associated with the risk of recurrent macrosomia [25]. Previous delivery of macrosomia was the single strongest individual risk factor for macrosomia controlling for prepregnancy BMI, excess weight gain, DM/GDM and other risk factors [22, 23]. In this study, previous delivery of macrosomia was also the most predominant risk factor for macrosomia in multivariate logistic regression analysis (OR 29.021, *P* < 0.05). Although this factor can not be controlled, it is helpful in screening for macrosomia.

The blood lipid level of pregnant women is one of the important factors affecting the birth weight of newborns. Maternal TG and TC can be taken up by the placenta, metabolized and transported to the fetus in various forms for providing energy for the fetus and helping the fetus to build cell membrane [26]. Elevation of these two lipids in a certain range during pregnancy are beneficial to the development and growth of the fetus. However, excessive lipid can cause fetal overgrowth [27]. Previous studies showed that maternal TG level in the first term of pregnancy was positively associated with higher birth weight, and an independent predictor of neonatal birth weight, but maternal TC level was not associated with birth weight [27, 28]. Whereas, Khaire A, et al. reported a positive correlation between maternal TC level and neonatal birth weight [26]. In this study, we found that the levels of maternal TC, TG in the first trimester in the macrosomia group were significantly higher than those in the non-macrosomia group (*P* < 0.05). The results of univariate logistic regression analysis showed the levels of maternal TC, TG in the first trimester were related to the occurrence of macrosomia. However, after adjusting for prepregnancy BMI, gestational weight gain, parity, history of macrosomia delivery, history of GDM/DM, HbA1c, and CRP, maternal TC level remained an independent risk factors of macrosomia in multivariate logistic regression analysis. Whereas, TG was not an independent risk factor for macrosomia.

HbA1c is a sensitive indicator reflecting glucose metabolism during pregnancy. Hatice Kansu-Celik, et al. have found a positive correlation between first-trimester HbA1c level and birth weight of newborns [29]. Similar to previous studies, we found that maternal HbA1c level in the first trimester was positively correlated with macrosomia and an independent risk factor for macrosomia, which can be used to predict macrosomia.

Previous studies have shown that increased CRP levels are associated with insulin resistance, maternal dysglycemia and GDM [30], which may cause macrosomia. In this study, first-trimester CRP levels were higher in the macrosomia group and related to macrosomia in univariate logistic regression analysis (*P* < 0.05). However, the result of multivariate logistic regression analysis showed that CRP was not an independent risk factor for macrosomia (*P* > 0.05), which was not consistent with the above findings.

Given the increased morbidity and mortality for infants and mothers caused by macrosomia, predicting macrosomia and taking effective interventions in early pregnancy were both crucial. Before 1976, fetal weight was estimated mainly by measuring the height of uterus and abdominal circumference of pregnant women. This method was easy and rapid, but it’s accuracy could be affected by many factors, such as uterine tension, amniotic fluid volume, fetal position and abdominal wall thickness. In recent 40 years, with the extensive use of ultrasound examination in obstetrics, ultrasonography has been currently an important measure to predict fetal weight in many countries. Sonographic estimated fetal weight (EFW) uses 2-dimensional ultrasound imaging to record fetal biometric parameters (such as abdominal circumference, head circumference, femur length, and biparietal diameter), and then a formula was entered to estimate fetal weight [31]. Compared with maternal physical examination, ultrasonographyis more accurate in assessing normal fetal weight. Whereas its sensitivity and specificity of evaluating macrosomia were only 0.56 (95% CI 0.49–0.61) [32] Although 3-dimensional (3D) ultrasound and magnetic resonance imaging (MRI) have been used to estimate fetal macrosomia, there is still lack of evidence to conclude that 3D ultrasound and MRI, which are more expensive, are superior to 2D ultrasound [33, 34]. Thus they are not widely used in clinical practice. Furthermore, these methods for predicting macrosomia are all carried out in the third trimester. Therefore, it requires to develop a simple, cheap and accurate method to predict the risk of macrosomia in early pregnancy.

Nomogram model has been widely used in clinical cohort studies due to its high accuracy, efficiency and stability. In this study, we constructed a nomogram model based on the risk factors of macrosomia including maternal BMI before pregnancy, parity, a prior macrosomic newborn, preexisting GDM/DM, the levels of HbA1 and TC in the first trimester. The AUC of the nomogram model indicated an overall predictive performance of 0.807 (95% CI: 0.755–0.859) in the internal validation, which showed good predictive ability. The sensitivity, specificity, positive predictive value, and negative predictive value were 0.716, 0.777, 0.174, and 0.977, respectively. Compared with traditional predictive models [35, 36], the nomogram model enables visual and personalized prediction, which can help obstetricians to more easily access the risk of macrosomia according to the score of each pregnant woman, then provide personalized healthcare service. Besides, the nomogram model can help pregnant women understand personal risk of macrosomia easily and clearly, which can enable pregnant women to cooperate with the intervention and achieve better clinical effects. In addition, the predictive model of this study is established based on maternal general characteristics before pregnancy and the clinical data in early pregnancy, which can be used to screen pregnant women for macrosomia in the early pregnancy stage so that effective intervention and treatment can be earlier implemented for these gestational women. More importantly, the variables of the screening tool are easily available, which is suitable for the wide promotion and application in clinical practice.

There were some limitations in the current study. The data was from a single center, the sample size was small, and the model was only internally validated. In addition, other demographic parameters, such as socioeconomic statu and genetic potential, were not considered, which were also associated with the macrosomia. Therefore, multi-center, larger sample studies with more parameters and further external validation should be carried out to explore the generalizability of this predictive model.

## Data Availability

The datasets used and analysed during the current study are available from the corresponding author on reasonable request.

## Abbreviations

BMI: Body mass index
GDM: Gestational diabetes mellitus
DM: Diabetes mellitus
HDP: Hypertensive disorders of pregnancy
SBP: Systolic blood pressure
DBP: Diastolic blood pressure
ALT: Alanine aminotransferase
AST: Aspartate aminotransferase
ALB: Albumin
TC: Total cholesterol
TG: Triglycerides
HDL-C: High-density lipoprotein cholesterol
LDL-C: Low-density lipoprotein cholesterol
SCr: Serum creatinine
UA: Uric acid
HbA1c: Glycosylated hemoglobin
FPG: Fasting plasma glucose
CRP: C-reactive protein
FT4: Free thyroxine
FT3: Free triiodothyronine
TSH: Thyroid stimulating hormone
